# Key elements and theoretical logic of live streaming e-commerce marketing discourse: An analysis based on grounded theory

**DOI:** 10.1371/journal.pone.0322495

**Published:** 2025-05-07

**Authors:** Haiyan Miao, Yongsheng Yin, Yuanyuan Zhao, Qian Gao

**Affiliations:** 1 School of Business Administration, Shandong Women’s University, Ji’nan, China; 2 School of Business, Shandong Yingcai University, Ji’nan, China; Federal University of Goias: Universidade Federal de Goias, BRAZIL

## Abstract

With the widespread adoption of mobile networks, live streaming e-commerce has experienced robust growth. Marketing discourse plays a crucial role in communication between live streamers and consumers. This study, adhering to the principles of typicality and diversity, selects 37 live streaming e-commerce videos and employs grounded theory for analysis. It explores the key elements of live streaming e-commerce marketing discourse and constructs a theoretical logic model. The study finds that product image portrayal, emphasis on service assurance, customer interaction, and live stream operation are key elements of this marketing discourse. Among them, product image portrayal is central, with its effectiveness dependent on the other three elements. Customer interaction enhances the “presence” of consumers, establishing a “reinforced quasi-social relationship” between streamers and consumers. Emphasizing service assurance effectively reduces consumers’ perceived risk. Live stream operation integrates these three elements to manage the live streaming process and consumers effectively. Based on these elements and the theoretical logic, the design of live streaming e-commerce marketing discourse can be more organized and effective, thereby positively influencing consumers and standardizing live streaming e-commerce activities.

## 1. Introduction

In the digital age, the important interactions between businesses and consumers are increasingly conducted through digital media and devices [1]. The integration of e-commerce and online live streaming has given rise to livestream e-commerce. Livestream e-commerce involves continuous real-time dissemination of product information through video, audio, and images to the public, commonly known as livestream selling. Here, livestreaming serves as a tool, and e-commerce forms the foundation [2]. The report titled “Observation on the Development Trends of the Live E-commerce Industry in 2024” released by the research team of the Institute of Finance and Trade Economics of the Chinese Academy of Social Sciences and the China Market Society, shows that from January to November 2024, the national live e-commerce retail sales reached 4.3 trillion yuan, contributing 80% of the incremental growth to the e-commerce industry [3]. At the same time, the ‘Live Commerce Platform Market’ report shows that the global live e-commerce platform market size was 918.9 million US dollars in 2023 and is expected to reach 53.9 billion US dollars by 2032, with a compound annual growth rate (CAGR) of 21.2% [4]. In this model, consumers can purchase products without leaving their homes, engaging in real-time interactions with sellers for a more appealing shopping experience and interpersonal connection [5]. Therefore, people are more likely to accept the consumption model in live streaming rooms [6].

Communication is indispensable for any marketing strategy [7]. In livestream selling, anchors use audience-oriented discourse, employing emotional involvement and price incentives to construct the livestream scenario. Under the influence of the anchor’s marketing discourse, audiences engage in a series of behaviors such as watching, following, and purchasing [8]. Thus, the effectiveness of an anchor’s selling capability is closely linked to their linguistic characteristics and discourse construction, particularly discourse with a strong personal touch, which tends to be more relatable and humorous, thereby garnering greater audience acceptance. This also makes the discourse of live-streaming marketing a topic worthy of study. Scholars have already begun to focus on the topic of live-streaming marketing discourse, but research remains relatively limited [9], mainly concentrating on discourse strategies and persuasive techniques [10], to serve the broadest research field of purchase intention in live streaming, thereby prompting consumers to make purchase decisions quickly. Huang et al. (2020) were the first to apply multimodal discourse analysis to deconstruct the discourse strategies in e-commerce live streaming [11]. Song et al. (2022) pointed out that the interactivity of live streaming can help achieve two-way and real-time communication between sellers and consumers, enhancing users’ perception of practical value in the context of fresh produce live streaming scenarios [12]. Lakhan et al. (2021) found that the entertaining situational atmosphere in live streaming rooms makes consumers trust the products more [13]. Cui (2021) highlighted the positive impact of the live streamer’s professionalism on consumer purchasing [14]. Gao and Liu (2022) focused on the “disciplining” and “micro-penalty” systems within the live broadcast room’s discourse [15]. Huang and Yang (2023) found that older hosts pay more attention to building rich emotional relationships with their audience. Previous studies have involved linguistic elements such as interactivity and entertainment [16]. Additionally, Chen Yi (2023) paid attention to the cultural attributes of the live broadcast discourse of the live streamer Dong Yuhui [17]. However, live-streaming e-commerce is a relatively new phenomenon, and there is no systematic scholarly review of the components of its marketing discourse system, nor has there been a clarification of the relationships between these elements.

To make livestream selling script planning more scientific and enhance the persuasiveness of marketing discourse, this paper explores the following questions: What are the key content elements of livestream marketing discourse? What are their interrelationships? What roles do they play in livestream selling? Base on this, this paper employs grounded theory to analyze anchor discourse in livestream videos, extracting key elements of marketing discourse, clarifying their logical relationships, and constructing a logical model of livestream marketing discourse. The hope is to provide theoretical references for the design of live-streaming sales marketing discourse, shifting the communication between anchors and consumers from homogeneous “shouting-style” [18] to a systematic approach.

The paper is divided into six sections. The remaining five sections are as follows: The second section is a literature review. The third section covers methods and data, detailing the research methods and data sources used. The fourth section presents the results analysis, including open coding, axial coding, and selective coding, with theoretical saturation testing. The fifth section is model interpretation and proposes strategies for constructing livestream marketing discourse. Section 6 is the discussion and conclusion, which introduces the main research findings, conclusions, and contributions of this paper, provides suggestions for the development of live commerce marketing discourse, and offers prospects for future research based on the limitations of the current study.

## 2. Literature review

Livestream selling is an emerging online shopping model that has recently gained traction primarily in Asia. Research on this phenomenon has mainly focused on consumer psychology and behavior or retail perspectives, with only a few scholars exploring livestream marketing discourse through discourse analysis [19]. To investigate the key elements and theoretical logic of livestream marketing discourse, two aspects of literature are relevant: (1) Livestream E-commerce; (2) Livestreaming E-commerce Marketing Discourse.

### 2.1. Livestream e-commerce

Currently, the definition of livestream e-commerce revolves around development background, components, forms, and characteristics [20]. A representative definition is that livestream e-commerce is a “reconstruction of people, goods, and venues,” where e-commerce platforms dynamically introduce, display, and recommend products via live broadcasts, creating a virtual online shopping scenario through real-time interaction to stimulate consumer desire and enhance shopping experience [21]. It has evolved into two main models [22]: embedded e-commerce live streams (e.g., Amazon Live, Taobao Live) and integrated e-commerce live streams (e.g., Facebook Live, TikTok). Both models exhibit characteristics of visibility, interactivity, and authenticity [12].

Regarding the impact of livestream e-commerce on consumer purchase intentions, researchers have employed theories such as the SOR model and perceived value theory. Factors stimulating consumers include: first, information interaction, where product personalization significantly influences marketing effectiveness and purchase decisions, and information quality, such as entertainment and credibility, positively affects consumer experience and trust, thus influencing purchase intentions [23,24]. Second, characteristics of livestream e-commerce offer a three-dimensional visual experience and real-time bidirectional communication between sellers and consumers, enhancing perceived value and trust [12,23]. Third, the more professional, authoritative, and well-known a anchor is, the higher the consumers’ trust in the product will be. Consequently, the perceived functional value of the product, such as quality, will be stronger, and the emotional value will also be enhanced, leading to greater interest in the product among consumers. [13,14]. Fourth, price discounts, which are a major attraction in livestream selling compared to other sales forms [18,25]. Fifth, a sense of presence, where livestreams, such as tourism live streams, can effectively increase trust and influence travel intentions [26].

### 2.2. Livestreaming e-commerce marketing discourse

Discourse provides a way to discuss specific topics and needs to be constructed to serve purposes. Livestream anchors need to use marketing discourse to build professionalism, credibility, and attractiveness, complete product explanations, and create virtual customer interactions to achieve livestream effectiveness [14]. Research on live commerce marketing discourse is primarily concentrated on discourse strategies and persuasive rhetoric.

#### 2.2.1. Discourse strategy.

The marketing discourse strategies in live-streaming commerce primarily serve the purpose of facilitating customer orders. Scholars have paid attention to various strategies. Huang et al. (2020) proposed strategies such as interjections, imperative sentences, personalization, and metaphors; [27]. Other scholars have focused on the significant role of interactivity. Song (2022) pointed out that interactivity can enhance users’ perception of utilitarian value [12]; Han et al. (2024) confirmed the positive impact of interactivity on consumers’ purchase intentions [6]. Some scholars have also paid attention to the positive experience entertainment brings to consumers [13]. Another important area is psychological strategies. Song and Mo (2024) found that Dong Yuhui mainly adopts strategies of disseminating knowledge, telling interesting stories, and using poetic language in his live discourse to meet customers’ intellectual, emotional, and psychological needs [28]. Zhang (2021) conducted an in-depth study of psychological strategies, employing theories such as guided consumption, decoy effect, reward effect, and authority effect to elucidate their roles in impulse buying [9].

#### 2.2.2. Persuasive rhetoric.

Discourse is a linguistic act aimed at achieving specific communicative purposes. ‘Persuasion’ is one of the objectives of live-streaming marketing discourse, which ultimately leads to the purchase decision. Gao et al. (2021) used the ELM (Elaboration Likelihood Model) to explain how consumers in live broadcast rooms process persuasive information and validated that central route factors such as message completeness and peripheral route factors such as the attractiveness of the host both have perceived persuasiveness [29]. Gong (2023) explored the impact mechanism of Taobao live marketing discourse at different stages on consumer cognition, emotions, and behavioral tendencies [30]. Chen and Zhou (2022) found that logic, professional knowledge, ethics, and emotional appeal are the main factors influencing the persuasiveness of anchors [31]. In the use of language tools, livestream discourse often employs diverse, novel, and polite vocabulary choices [32], supplemented by rhetorical devices such as metaphors and personifications [27,33,34],to create guiding, emotional, and social discourse forms[35], which in order to achieve the purpose of persuasion. However, vocabulary and theme choices often display gender differences [36].

Scholars have also noted ethical issues in marketing discourse, such as dishonesty. Anchors may adopt a “victim” identity to gain sympathy or use inflammatory language to trigger impulsive purchases [37]. Additionally, assertive, confident and euphemistic speech all influence consumers’ objective judgment [38].

### 2.3. Review

Research on live-streaming marketing discourse is currently in its infancy, with limited attention paid on the multimodal, highly interactive nature of livestream e-commerce marketing discourse. Existing studies have mainly focused on discourse strategies and persuasive techniques, and has not yet been a systematic study and discussion on the constituent elements of the content of livestream marketing discourse. This gap hinders the guidance on how live-commerce marketing discourse can form an integrated chain through structural symbolic interaction. Consequently, it fails to guide the new integrated communication activities empowered by technology in live-commerce to achieve the intersection of material identification, idealized identification, and formal identification for consumers [39]. It is even more challenging to generate consumer behavior under supportive emotional identification.

Based on this, the study aims to systematically analyze livestream marketing discourse. On the one hand, improving the communication effectiveness of livestream marketing discourse requires a systematic theoretical framework [40]. This framework should cover the key elements of livestream marketing discourse and clarify their interactions and content. On the other hand, theoretical guidance for practice needs to derive actionable measures from typical practices. Therefore, this study comprehensively collects and analyzes typical livestream videos, using grounded theory to summarize key elements of livestream marketing discourse and construct a theoretical model.

## 3. Research design

### 3.1. Research method

Grounded theory emphasizes exploring and constructing social phenomena inductively in natural settings. Through in-depth analysis of qualitative data, it effectively reveals the structure and causal relationships of events [41]. Grounded theory is a research strategy that aims to construct a rigorous theoretical foundation by adopting a bottom-up approach, systematically inducing core concepts related to the research subject from empirical data. It bridges the gap between raw data and research findings, providing researchers with a complete set of methods and steps to inductively derive and construct theories from primary data. Although livestream selling has developed rapidly, it is still relatively new, and the field lacks mature theories. Grounded theory has significant advantages in constructing theories and is one of the more scientific methods in qualitative research. Therefore, this paper adopts grounded theory to systematically code and analyze video data of livestream selling, thereby systematically summarizing and logically refining the key elements of livestream marketing discourse. This approach is feasible for addressing the research questions of this study.

**Fig 1:**
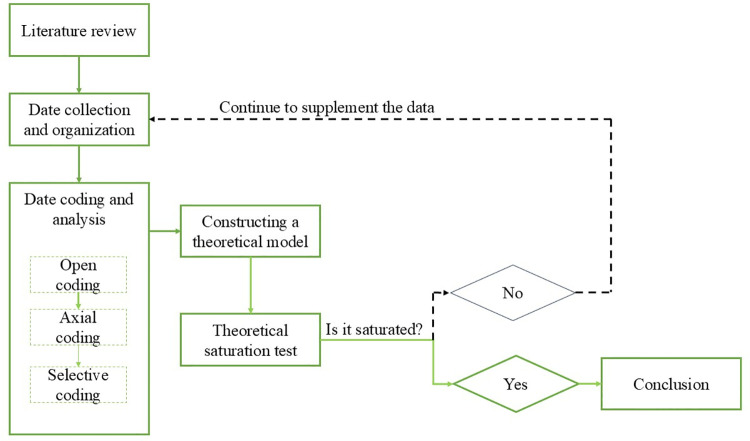
The research flowchart of grounded theory.

Fig 1 illustrates the research process. Based on the research objectives, the study formulates questions and conducts a systematic literature review. Then, grounded theory is applied to organize and analyze the livestream selling video data. Following transcription of the videos, the research proceeds with open coding, axial coding, and selective coding. After passing the theoretical saturation test, a theoretical model of the key elements of livestream marketing discourse is constructed.

### 3.2. Sample selection and data collection

Research based on grounded theory should follow the principle of theoretical sampling [41]. The representativeness of the samples, determined by their typicality, is crucial for meeting research needs [42]. The data for this study comes from livestream selling videos. The sample selection adheres to the following principles:

First, according to the research questions and considering sample typicality, the sampling range is limited to account video data from platforms like Taobao, TikTok, and Amazon Live, such as the Li Jiaqi Austin livestream room. Second, diversity in video data is considered. The selected videos feature different types of livestream anchors and products. The anchors include males and females, internet influencers, merchant streamers, government officials, and ordinary individuals. The products cover various categories, including food, beauty, clothing, daily necessities, agricultural products, jewelry, home appliances, travel, toys, and educational training. Therefore, the selected samples possess typicality and diversity, adequately addressing the research questions.

A total of 37 livestream videos featuring 37 different anchors were collected for this study. The videos’ audio was transcribed into text using iFLYTEK Hearing software, resulting in nearly 90,000 words. The sources and basic information of the samples are detailed in Table 1. The sample size was determined based on product types and demographic characteristics of the anchors, with multiple rounds of sampling conducted according to the principle of theoretical saturation.

**Table 1:** Sample Characteristics Information.

Characteristic	No. of Participants	Percentage
**Live streaming platform**		
Tik Tok	12	35.135%
Taobao	15	43.243%
WeChat	5	16.216%
Amazon Live	5	5.406%
**Gender of anchor**		
male	20	54.545%
female	17	45.455%
**Anchor identity**		
Professional anchor	18	48.485%
Cross-border anchor	11	27.273%
Amateur anchor	8	24.242%
**Product categories**		
Packaged food	3	8.108%
Non-staple food	2	5.405%
Grain and oil	2	5.405%
Fresh food	4	10.811%
Office supplies	4	10.811%
Hardware and home appliances	4	10.811%
Washing and daily chemicals	5	13.514%
Knitted clothing	8	21.622%
Jewelry, jewelry	3	8.108%
Domestic services	2	5.405%

In the first round, 20 samples were selected based on product categories, including clothing, gold jewelry, home appliances, skincare products, online courses, fast food, travel, fresh agricultural products, and toys. In the second round, considering the gender of the anchors, one additional sample each was selected for home appliances, clothing, agricultural products, and daily necessities, totaling 4 samples. In the third round, considering the identity of the anchors, one sample each was added for ordinary individuals, government officials, actors, and corporate executives, totaling 4 samples.

In total, 3 rounds of coding were conducted, with 28 samples selected. The coding analysis indicated that theoretical saturation was nearly achieved by the second round.

## 4. Construction of the key elements model of livestream marketing discourse

### 4.1. Open coding for concept and category extraction

Open coding involves coding, labeling, and logging the raw data word by word and sentence by sentence to develop initial concepts and extract categories from the original data [43]. Glaser (1998) proposed that during the process of using grounded theory, we need to maintain an open and free mindset to discover the roots of phenomena and deeply understand the connotations of concepts, and to conduct systematic analysis of data related to research questions in order to make open coding more effective [44]. This study uses the “iFLYTEK Hears” software to transcribe the collected video data into text. Based on this text material, the coding process at this stage is divided into three steps: First, this study selected 20 out of 37 original documents for data decomposition, and extracted 269 original statements from all the content based on objective judgment and comprehensive understanding. The second step is to develop the initial concepts. The nodes formed by initial coding are continuously compared, analyzed, and summarized, and 98 initial concepts are derived through merging and organization. For example, when multiple anchors mentioned the issue of following the account, we categorized it as one, coding it as “Invitation”. The third step is to extract categories. Categories are further refined from concepts and are formed by clustering multiple concepts with the same orientation. For example, “greetings,” “compliments,” and “thanks” all serve to reduce communication friction and are related to the smooth use of language in interactions, which are summarized into the category of “polite language.” In order to ensure the reliability of the coding process, it is first independently completed by one researcher, then verified by another researcher. Finally, the research team conducts multiple discussions based on the frequency of concept occurrence and the redundancy among concepts. In the end, 15 initial categories were obtained (Table 2).

**Table 2:** Open Coding.

Initial Category	Concept	Original sentences from the video material
F_1_Basic knowledge of products	f_11_ product name	The first one is Jeep’s outdoor sun hat. Today we bring you two styles, including our baseball cap style.
	f_12_ function	The explosive body cream sold in our live broadcast room has both sun protection and whitening properties, and it is a body cream for all girls to brighten their skin tone.
	f_13_ brand	Now I`m going to introduce to you the jujubes of Hao Xiang Ni from Xinjiang.
	……	
F_2_ Product source information	f_21_ location	The central and southern parts of Shandong are basically very far south. The Taishan area and the Yimengshan area have very high altitudes, and the peaches grown there are very sweet.
	f_22_ live room name	Video Hello, everyone from CCTV News, welcome to watch. We are the “Strive for a Better Life” online live broadcast program jointly launched by CCTV News and Gome.
	f_23_ inventory	Yams come from this small alluvial plain at the corner of the Taihang Mountains and the Yellow River.
	……	
F_3_ product appearance	f_31_ color	This is an extra-large pink bottle, which sells particularly well in the live broadcast room. It is a combination of Yuanqi shampoo and conditioner.
	f_32_ types	This is for two consecutive nights, you can choose between a king-size bed and twin beds, and it is a business suite. The normal price for a single bed is more than 2,000.
	f_33_ size	Look at the book which is about the size of a palm.
	……	
F_4_ Product Features	f_41_ uniqueness	Now you can’t enter Tsinghua University and Peking University without a student card. You can only enter through a research program like ours.
	f_42_ personnel value	Including me, there are 8 teachers from Tsinghua University’s Computer Science Department in our team. 3 of them were admitted to Tsinghua University’s Computer Science Department by ranking among the top in the college entrance examination. The remaining 5 teachers were directly admitted to Tsinghua University’s Computer Science Department through the most prestigious programming competition we just mentioned.
	f_43_ convenience	When you are hungry, just make a cup. It’ll be ready in five minutes.
	……	
F_5_ Commodity Storage	f_51_ shelf life	All girls have a shelf life of 270 days, so don’t worry.
	f_52_ save method	Freeze the bayberries that are in abundance in June, and you can eat them in different seasons.
	f_53_ reuse	The replay of this live lesson will be released immediately to everyone on the same day.
	……	
F_6_ Product Price	f_61_ price	Let’s look at the price first. It’s about the same price as another product we sell, and it contains 10 grams more per bag. The price is also 99, and there are 5 bags.
	f_62_ price and timeliness	The exclusive discount for 521 Valentine’s Day is today, the last day.
	f_63_ price comparison	Our products have the same price as the other one, but 10 grams more per bag.
	……	
F_7_ Business Promotion	f_71_ cash gift	We are giving away 20 lucky bags, each of which comes with a 10-yuan voucher.
	f_72_ discount	If we fill in the quantity of three, we will get a 56-yuan coupon and the final price will be 121 yuan.
	f_73_ bonus item	You will receive four packs of laundry detergent and a double-layer steamer. The backstage staff will make a note directly.
	……	
F_8_ Politeness	f_81_ greetings	Hello, hello, everyone, happy new year.
	f_82_ praise	The young lady has a good eye.
	f_83_ thank-you phrases	Thank you all for your trust in me.
	……	
F_9_ Relationship Building	f_91_ invitation	If you haven’t followed us yet, please click the follow button in the upper left corner of our screen. Become our family and let us spoil you.
	f_92_ resonates	Life will improve. I couldn’t even eat chicken at the beginning, but today I am selling chicken here.
	f_93_ ask for help	Could you do me a favor and leave more comments?
	……	
F_10_ Personalized Suggestions	f_101_ purchase advice	You can buy one box of each of the three flavors, or if you are buying it specifically for children, you can buy three boxes of a single flavor.
	f_102_ usage suggestions	Let me tell you the reading order of the four masterpieces. First read Journey to the West, then read Water Margin, then Romance of the Three Kingdoms, and finally Dream of the Red Chamber.
	f_103_ operational guidance	If your shopping cart experiences lag, don’t worry, just keep clicking repeatedly.
	……	
F_11_ Logistics Services	f_111_ express partner	The first 30 people who place orders today will have their orders shipped today, using SF Express for air transportation.
	f_112_ delivery range	We cannot ship to Hainan, but we can ship to other regions normally.
	f_113_ dispatch guidelines	Please let me know if you’ve placed an order, and I’ll arrange for expedited shipping for you.
	……	
F_12_ After-sales Service	f_121_ return and exchange	If you don’t like it after receiving it, please contact customer service to help you.
	f_122_ maintenance cost	No matter which product you buy, as long as it has the Gree logo, it will be free for 10 years of maintenance.
	f_123_ post-purchase guide	Click on the small yellow cart below and open any product. In the lower left corner, there is a customer service icon.
	……	
F_13_ Live Rhythm Control	f_131_ customer accumulation	Friends who want to join the summer camp can wait in the live broadcast room, we will prepare the link in the background.
	f_132_ reminder	There are 7 orders left. The first 30 people who place their orders will have them shipped to you today.
	f_133_ repetition	Welcome, new friends! Let me introduce again.
	……	
F_14_ Live Preview	f_141_ single product quantity	I have prepared 18 fashion items today. Time is a bit tight, so I will just give you a quick preview.
	f_142_ sales time	Link No. 1 only gives you two minutes
	f_143_ broadcast start time	Feng Feng’s live stream time on the book channel should be 6:00 p. m. tomorrow. You can set a clock.
	……	
F_15_ Live Purchase Rules	f_151_ kick order rules	If you don’t pay for more than half an hour, I will automatically assume that you don’t want it, okay?
	f_152_ welfare rules	If you want it, please type the word “want” and the anchor will give you a bonus.
	f_153_ others rulers	There are 30 lucky bags at the upper left corner on the screen, you can click on it. They will last for 10 minutes.
	……	

### 4.2. Axial coding

The task of axial coding is to develop the properties and dimensions of categories and discover the potential logical relationships between them, thereby developing main categories and their subcategories [41]. The main task of axial coding is to identify the commonalities between concepts and establish linkages between their attributes, fostering interconnections among various dimensions so that all content can be organically integrated. Based on the correlation and logical relationships among different categories within the concepts, the concepts and categories extracted from open coding are further deeply analyzed for each category. Concepts that cannot be grouped with others into categories are eliminated. We need to repeatedly read the materials related to the categories, check for the emergence of new concepts, and ensure the exclusivity and rigor of the categories.

This study explores the “key elements and theoretical logic of livestream marketing discourse.” Based on the logical internal connections of different categories at the conceptual level, they are classified and summarized into four main categories. The main categories and corresponding initial categories are shown in Table 3. The elements such as name, appearance, price, and features, which are related to product introduction, are summarized into the main category of Product Image Portrayal (Z1). Polite language, relationship building, and personalized suggestions, which aim to engage consumers and promote two-way communication, are classified under Customer Interaction (Z2). Logistics and after-sales service, which are the “behind-the-scenes” service processes of the livestream, are categorized into Emphasizing Service Guarantees (Z3). Livestream pace control, livestream announcements, and livestream purchasing rules, which are part of the management work during the livestream process, are grouped under Operations During Livestreaming (Z4).

**Table 3:** Main Axis Coding.

Main Category	Corresponding categories	Category Connotation
Z1 Product Image Characterization	F1 Basic knowledge of products	Basic product knowledge is to define the commodities in the live broadcast from the dimensions of product name, brand, function, value and usage, and to explain the scope of application from the objects of use and scenarios.
	F2 Product source information	Product origin information refers to the situation of the sellers in the live broadcast room and an introduction to the place of origin from the perspectives of geographical location, ecology and cultural environment.
	F3 product appearance	Product appearance refers to the visual appearance of the product, including the shape, pattern, color, structure, volume, model and other aspects of the packaging, and is also the type of specifications that consumers can choose.
	F4 Product Features	Product features refer to the advantages that a product has beyond its basic functions compared to its competitors. They are usually manifested in quality features such as stability and reliability, and appearance design that bring consumers a good user experience.
	F5 Commodity Storage	Storage refers to consumers properly preserving goods so that they can continue to be used within their shelf life.
	F6 Product Price	Price refers to the selling price of the goods in the live broadcast room, which comes in three forms: the original quotation in the shopping cart, the unit price in the live broadcast room, and the actual paid price under the business promotion discount.
	F7 Business Promotion	Business promotion refers to preferential measures formulated for live broadcast products under specific sales situations, which are often manifested as price discounts or gift giving.
Z2 Customer Engagement	F8 Politeness	Polite language refers to the language used by live-streaming hosts to express gratitude, respect, care, praise, etc. when interacting with customers.
	F9 Relationship Building	Relationship building refers to the timely and continuous interaction between the live broadcast host and the customers based on the shaping of their own and the customers’ identities, using the live broadcast room functions (host’s language expression, attention, public screen interaction, gift sending, likes), to build an increasingly close relationship, so that customers can receive information in real time, express demands, resonate with cognition, evaluate after purchase, and recommend purchases.
	F10 Personalized Suggestions	Personalized suggestions refer to the anchor’s answers to various questions that consumers encounter when purchasing products in the live broadcast room and in subsequent use based on his or her own experience and professional knowledge.
Z3 emphasizes service guarantee	F11 Logistics Services	Logistics service refers to the process and method of the flow of goods in the live broadcast room from the shipping point to the customer under the constraints of commodity inventory quantity and express delivery conditions.
	F12 After-sales Service	After-sales service refers to the commitments and explanations made by the anchor regarding who customers can turn to, as well as the content, level and methods of service available.
Operations in Z4 Live	F13 Live Rhythm Control	Live broadcast rhythm control refers to the live broadcast host following the script design to reasonably arrange the time slots for each product and the recommendation process of a single product.
	F14 Live Preview	A live broadcast preview refers to the host’s description of the product catalog, time schedule, and other live broadcast sessions.
	F15 Live Purchase Rules	Live broadcast purchase rules refer to the rules that customers need to follow when participating in live broadcast purchases to satisfy their personal needs of obtaining information, grabbing orders, enjoying preferential treatment, and getting help. They are usually manifested in time limits and task completion.

The four main categories are interrelated. For instance, product price belongs to Product Image Portrayal, but the final amount paid by consumers also depends on logistics costs, purchasing rules, and the participation in interactive activities under those rules during the livestream.

### 4.3. Selective coding and model construction

Selective coding involves categorizing and abstracting main categories into core categories by exploring the intrinsic relationships among them [41]. The main purpose of selective coding is to select the “core category,” deal with the relationships between categories, and form a synthesis process. This process involves clarifying the primary and secondary among categories, establishing connections between the core category and secondary categories, and clarifying the entire “storyline” to form a new substantive theoretical framework related to the theme. This study identifies livestream marketing discourse as the core category. Surrounding this core category, the “storyline” structure is as follows: product image portrayal, emphasizing service guarantees, customer interaction, and operations during the livestream are all organically integrated parts of the content of livestream marketing discourse, presented according to a predetermined script logic.

Product image portrayal is the real-time dynamic display of product information and is the core of livestream marketing discourse. Customer interaction enhances consumer engagement through scenario-based interactions. Emphasizing service guarantees acts as a quality endorsement from a supply chain perspective, supporting the effectiveness of product image portrayal. Both service guarantees and customer interaction are powerful supports for the effectiveness of product image portrayal. Operations during the livestream permeate these three elements, achieving comprehensive management of the livestream process. These four elements are intertwined and integrated, forming the key elements of livestream marketing discourse.

**Fig 2:**
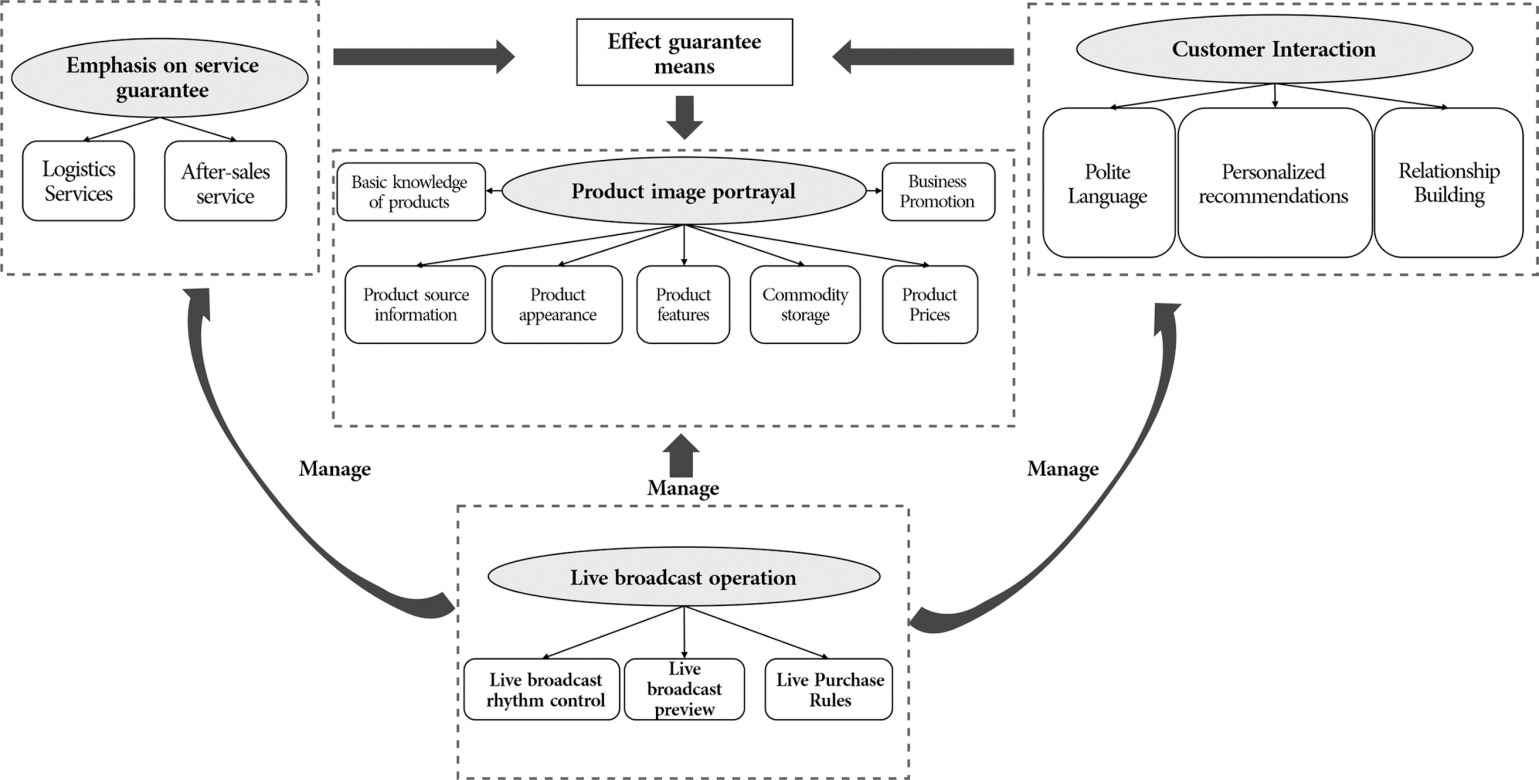
Theoretical logic model of key elements of live streaming marketing discourse.

By coding the text data derived from livestream videos, the study constructs a complete “storyline” centered around “livestream marketing discourse” and builds a theoretical logical model of the key elements of livestream marketing discourse (Fig 2).

### 4.4. Theoretical saturation test

To ensure the scientific rigor and accuracy of the grounded theory research process and results, this study first conceptualizes the implicit interrelationships between the concepts or categories formed through open and axial coding. Subsequently, the initially constructed theories and concepts are continuously compared with existing literature and concepts, resulting in no new conceptual dimensions emerging. Therefore, from a theoretical and conceptual perspective, the coding has reached saturation. Furthermore, a theoretical model saturation test was conducted using an additional 9 sample materials. The coding analysis of these 9 samples revealed that all were encompassed by the previously extracted four main categories. Thus, this study concludes that the preliminary selective coding established is theoretically saturated.

## 5. Key elements and model explanation of livestream marketing discourse

This study uses grounded analysis on livestream selling video data to construct a theoretical logical model of the key elements of livestream marketing discourse (Fig 2). The model involves four key elements: product image, service, customer interaction, and operations, with their logical relationships and dimensions explained as follows:

### 5.1. Product image portrayal

Product image portrayal (basic product knowledge, source information, appearance, features, price, promotional activities, and storage) is the core of livestream marketing discourse, where anchors represent consumers in product testing to convey physical attributes. According to studies on consumer psychological contracts in livestream scenarios, the development of transactional psychological contracts requires anchors to help consumers select high-quality and unique products and services [45]. Establishing a contractual relationship first requires meeting consumers’ utilitarian motivations [24]. In scenarios where product factors act as central routes [46], product quality and discount information are most likely to attract consumer attention.

Basic product knowledge involves the anchor introducing product functions and applicable ranges. Functional utility is a primary prerequisite for achieving a contractual relationship. Based on involvement theory, creating useful consumption scenarios can increase consumer involvement [47], enhancing the persuasive effect of marketing discourse. Product source includes the seller in the livestream and the place of origin, which are guarantees of product utility. According to signaling theory, anchors reduce information asymmetry through information exchange, endorse product quality via reputation effects, and signal supply chain management capabilities [48], thereby gaining consumer trust. Additionally, the geographical location, ecology, and cultural environment of the origin support the product’s functional value. Such information conveys positive signals about supply chain management, reinforcing consumer trust in the livestream’s supply chain capabilities.

Product appearance, observable through visuals, includes dimensions such as color, pattern, structure, and model, making product image portrayal concrete. Product appearance is the physical attachment of product functions, combining visibility and aesthetic appeal, which positively impacts purchase intentions [49]. Product features are advantages beyond basic functions, often manifested as stability, reliability, and aesthetic design, offering a superior user experience and central route persuasion. This information, belonging to experience goods characteristics [48], helps consumers compare different products, influencing satisfaction, post-purchase evaluation, and repeat purchase rates.

Storage extends the utility of products over time, as in “freezing June-harvested bayberries to enjoy them in different seasons,” demonstrating the temporal extensibility of product use value. From a supply chain perspective, anchors guide consumers from shopping scenarios to usage scenarios, especially for perishable goods [12]. Price, a key component of consumers’ monetary costs, is a central route information like product features. In livestreams, price information often combines with promotional activities to stimulate utilitarian motivation, such as “fill in three quantities to get a 56-yuan coupon, bringing the final price to 121 yuan,” making the actual price lower than the cart price and significantly encouraging purchases.

In summary, the research shows that anchors should first establish a “benefit relationship” between the product and the consumers when portraying the product image, with the focus of this relationship being on quality and price. As Shang et al. (2023) found in their research: the product-background match affects consumers’ perceived value, which in turn influences their emotions and behaviors [50]. Anchors must be well-informed about product details and provide truthful, comprehensive introductions, allowing consumers to perceive a “transactional norm.”

### 5.2. Customer interaction

Polite language, relationship building, and personalized suggestions are the main interaction methods between anchors and customers in livestream selling. Customer interaction ensures the effectiveness of product image portrayal by creating a scenario atmosphere and establishing emotional connections. Scholars have proposed the concept of one-way, non-dialogical parasocial interaction [51]. In livestream selling, parasocial interaction is strengthened and integrates reciprocity and fan culture, termed “enhanced parasocial interaction.” This interaction enhances consumers’ social presence [52], positively impacting online purchase intentions. Compared to parasocial interaction, “enhanced parasocial interaction” has the following characteristics: First, it exists based on the extensive use of social media. Social media provides the technology and platform for interaction, which traditional media such as television and radio do not possess. Second, with the help of social media’s information reply and live chat technologies, its interaction is bidirectional, while parasocial interaction has a certain degree of illusory nature; Third, audiences are not only selectors of relationships but also creators of relationships, thus having greater autonomy. Fourth, the audience can interact with each other. Therefore, as Yuan and Gao (2020) pointed out, “enhanced parasocial interaction” makes the audience perceive the social presence and the authenticity and approachability of media figures [53].

Polite language in livestream selling expresses greetings, compliments, and thanks, forming a universal interactive approach. Polite language reinforces consumers’ perception of emotional care responsibilities, as seen in phrases like “You have a great eye,” which serve as persuasive tools. Broad use of polite phrases like “Happy New Year” can effectively reduce psychological distance in virtual spaces, enhancing customer commitment. Highly committed consumers are more likely to trust long-term stable transaction relationships in livestreams [54], promoting both transactional and relational psychological contracts between consumers and anchors.

Relationship building involves timely, continuous interactions between anchors and customers using livestream functions (e.g., language expression, follow requests, screen interactions, gift-giving, likes), constructing increasingly close relationships. This interaction aims for real-time information reception, expressing demands, cognitive resonance, post-purchase evaluation, and recommending purchases, adding value through extensive interaction. The dramaturgical theory, developed from symbolic interaction theory, explains anchors’ and consumers’ interaction behavior in livestreams [55]. Research indicates that anchors actively engage in symbolic interaction by inviting consumers to “click the follow button in the top left corner,” hoping to become “family.” Consumers also actively participate in symbolic interaction through actions like gifting (e.g., lollipops on WeChat and TikTok livestreams), seeking the anchor’s attention and reflecting significant symbolic interaction empowered by technology. Relationship building enhances consumers’ sense of presence, including coexistence, communicative, and emotional presence [52], leading to higher information trust.

Personalized suggestions involve anchors offering advice based on their expertise and professional knowledge to address various issues consumers face when purchasing or using livestreamed products. Studies find that anchors interact with customers using specific “pragmatic identities,” collectively termed “anchor pragmatic identities.” Advice like “let me tell you the reading order of the Four Great Classical Novels” reflects the anchor’s professionalism, which positively influences purchase intentions [56]. Personalized suggestions are highly interactive, as seen in advice like “if you’re buying for kids, get three boxes of a single flavor,” enhancing customers’ social presence. Based on social facilitation theory [57], increased social presence can also trigger conformity consumption behavior.

### 5.3. Emphasizing service guarantees

Emphasizing service guarantees extends the “consumption scenario” to the “post-purchase scenario,” strengthening trust to ensure the effectiveness of product image portrayal. With the help of information technology, the backstage that is invisible to the audience in dramaturgical theory gradually becomes known to consumers in live commerce, which can be termed as the “semi-open backstage.” The “semi-open backstage” refers to the real-time and dynamic presentation of pre-sales and after-sales information. For instance, in agricultural product live streaming, the fields where crops are grown act as the live broadcast room. Additionally, consumers can track shipping and logistics information in real time. As Xiong (2021) pointed out, these practices reduce the hierarchy in mass communication and the imbalance in information resources. At the same time, anchors need to guide consumers in using this backstage information effectively [48]. At the same time, the scope of the livestream “crew” extends beyond the livestream room, encompassing the entire supply chain, called the “extended livestream crew.” New crew members joining must undertake “script tasks.” For example, the information display of logistics service providers has a persuasive effect, while comments from other customers serve as a reference [58]. This requires anchors to have strong on-site organizational and guiding abilities. Logistics and after-sales services are critical backstage performance aspects. Increased information transparency and process control reduce perceived risks in contractual relationships during “enhanced parasocial interaction,” enhancing purchase intentions [59].

Logistics service involves the movement and process of livestream products from dispatch points to customers, constrained by inventory and shipping conditions, encompassing three dimensions: inventory, shipping arrangements, and courier partners. Logistics service provides external support for the persuasive effect of livestream marketing discourse. Based on the theory of reasoned action [60], consumers’ logistics expectations focus on timeliness, reliability, and promptness. Firstly, based on disciplinary theory [61]regarding inventory, anchors often use disciplinary language like “only one left on link one” to construct a “punishment” scenario of price recovery or product sellout, taming consumers. Although consumers recognize this [37], they cooperate due to utilitarian motivation. For shipping arrangements, anchors explain shipping order rules and arrangements, promoting ordering through orderly construction. Consumers’ compliance with order is driven by their desire for “rewards.” Finally, courier partners are key members of the “extended livestream crew,” where phrases like “ SF Express and air transport” highlight the leverage of the “ SF Express” brand on livestream products.

After-sales service involves anchors’ commitments and explanations about assistance, service content, service levels, and methods available to customers, providing internal support for the persuasive effect of livestream marketing discourse. Online merchants’ reputation levels are crucial purchasing reference information [62]. Comprehensive after-sales service is vital for maintaining reputation and ensuring trust, adhering to transactional norms, and maintaining relationships, continuously strengthening the psychological contract between consumers and anchors and promoting the development of their relationship lifecycle.

### 5.4. Operations during livestreaming

Anchors must perform operational management duties such as controlling the livestream pace, announcing livestream activities, and regulating purchasing rules. Operations during livestreams often permeate product image portrayal, customer interaction, and service guarantees, ensuring the livestream proceeds as planned. Research finds that while actively constructing contractual relationships with consumers, anchors discipline and punish “trainees” through livestream behavior rules, establishing power relationships. Anchors combine “commands” and “rewards and punishments” to create community discourse within the enclosed livestream space. To achieve disciplinary goals, power operations often use discipline and enclosed space techniques.

Controlling the livestream pace involves anchors arranging each product’s time slot and the recommendation process for individual products according to the script. At the micro level, anchors use “discourse rhythm discipline,” like “buy it! buy it!” short phrases to immerse consumers in the temptation of cheap product prices and numerous gifts. As Gao and Liu (2022) pointed out, the tension and rhythm created by speech speed inhibit consumer thinking, making them act according to instructions [15]. At the macro level, organizing orderly product sales processes according to the script benefits consumer experience. Therefore, pace control usually combines with guiding consumer behavior. The presence of multiple people enhances customer sensory experience [62], stimulating purchase intentions.

Announcing livestream activities involves anchors explaining the livestream product list, time schedule, and other session details. According to psychological ownership theory, announcing livestream information enhances customers’ sense of control, generating morepositive emotional responses [63]. However, anchors also emphasize the limited time for single product availability to prompt purchases, such as “only two minutes for link one.” This highlights the strategic differences at macro and micro levels, resulting in “incomplete psychological ownership” for consumers.

Livestream purchasing rules are the guidelines customers follow to gain information, secure orders, enjoy benefits, and receive assistance during livestream purchases. These rules often present as time limits and task requirements for consumer discounts. Livestream purchasing rules test and facilitate the effect of livestream marketing discourse, forming a closed-loop process of “symbolic rules-output-symbol recognition-input” between anchors and consumers. Consumers need to “follow to get quotes,” showing that satisfying consumer desires requires recognizing the livestream’s symbolic representations and extended “rules.” Recognizing rules equates to recognizing the product, smoothly transitioning into the consumption phase.

### 5.5. Construction strategies for livestream marketing discourse

Based on dramaturgical theory, parasocial relationship theory, discipline theory, and psychological ownership theory, constructing marketing discourse should focus on “audience expectations,” showcasing the power of the “extended livestream crew,” promoting “psychological contracts” through “enhanced parasocial interaction,” and reducing consumers’ perceived risk of “anchor control” to enhance their sense of control. Specific strategies include:

(1) Understanding Audience Expectations and Setting Plot Content: According to dramaturgical theory, anchors work according to a scripted “plot,” which should be set based on accurately grasping “audience expectations.” These expectations include their information needs and role expectations for the anchor. Rational information like product quality and price can achieve central route persuasion through utilitarian motivation, while anchor professionalism and attractiveness influence purchase intentions through hedonic value. Overall, objective presentation of quality and price information should be based on high professional skills and integrity.
(2) Showcasing Crew Strength and Enhancing Parasocial Interaction: Following the set “plot rhythm,” the “crew” continues various forms of “enhanced parasocial interaction,” creating a stronger sense of presence, accessibility, recognition, and emotional involvement for consumers compared to traditional parasocial relationships. This helps achieve “transactional” and “relational” contracts. Meanwhile, anchors should coordinate with crew members to showcase their strength, simplifying the purchasing process and reducing consumer information processing difficulties.
(3) Enhancing Social Responsibility of Anchor Control to Boost Consumer Control: Foucault’s discipline theory [61] posits that power holders can use discourse to influence audiences to meet their expectations in both actions and methods. Anchors can use tools and techniques under social responsibility constraints to achieve disciplinary consumption behavior. They can establish order and reward mechanisms while using vocabulary and speech pace to control rhythm, achieving relationship building and promoting transactions. Simultaneously, anchors should enhance consumer psychological ownership to ensure emotional value is obtained.

## 6. Discussion and conclusions

### 6.1. Discussion

Research has found that product image portrayal is the core content of live commerce marketing discourse, particularly information about competitive pricing and quality. This aligns with the findings of Song and Mo (2024) and Shang et al. (2023) [28,50]. Product image portrayal helps achieve background consistency between consumers and the product setting, serving the fundamental purpose of consumers. Of course, this process requires hosts to possess a high level of professionalism and the ability to gain trust. Previous research has indicated that competence, benevolence, and integrity are the three key elements in building trust. As Huang et al. (2020) found: Li Jiaqi often criticizes the products he tries in live broadcasts, which makes people perceive him as candid and genuine [27]. Of course, making boring product information interesting is very important. This study specifically mentions the integration of entertainment and cultural elements. As Song and Mo (2024), Chen and Zhou (2022) and Shang et al. (2023) have pointed out: perceived pleasure and cultural integration play a role in promoting consumer purchases [28,31,50].

Based on the unique characteristics of live commerce, this study proposes the concept of “enhanced parasocial interaction” on the foundation of the original parasocial interaction theory. The research suggests that there are significant differences between enhanced parasocial interaction and traditional parasocial interaction in terms of information transmission pathways, relationship building, and interaction effects, which aligns with the findings of Yuan and Gao (2020) [53]. In this model, consumers have greater say and choice in communication, becoming creators and disseminators of information. Particularly, relationship building requires the live commerce team to place a strong emphasis on attracting consumer attention and managing their emotions. Song and Mo (2024), Shang et al. (2023), and Shi and Dou (2023) all highlight the importance of attracting consumers and building harmonious relationships in their research [28,50,64].

Based on dramaturgical theory, this study includes members and information from the supply chain in its research scope, proposing the concepts of “semi-open backstage” and “live commerce extended crew.” This aligns with the research of Xiong et al. (2021), which emphasizes that the information exchange mechanism positively affects consumer trust by reducing information asymmetry [48].

Research has found that while live commerce builds harmonious relationships with consumers, it also implements discipline. Discipline is primarily achieved through discourse and rules. As studies by Gao and Liu (2022), Gong and Jin (2023) have pointed out: persuasive language such as order-pressing and novel metaphors have strong persuasive power [15,30]. This is especially evident when it is linked to consumer interests. For example, Li Jiaqi describes the feeling of applying lipstick by comparing it to familiar and imagined foods that the audience knows.

### 6.2. Research conclusions

The rapid development of live commerce has been accompanied by a nascent stage in the research of its marketing discourse. Recognizing the significant role of marketing discourse, this paper focuses on exploring the following questions: What are the key content elements of live streaming commerce marketing discourse? What are the relationships between these elements? What roles do these elements play in live streaming commerce? The study employs grounded theory methodology, using live commerce videos as primary data. It conducted a step-by-step coding and systematic analysis of 37 transcribed live commerce video materials, identifying the key elements of live commerce marketing discourse and constructing its theoretical logic. The specific conclusions drawn are as follows:

(1) Livestream Marketing Discourse: The primary communication tool used by anchors in livestream selling is marketing discourse. Through the application of this language tool, anchors design and execute product demonstrations, customer interactions, and internal operations management according to predetermined steps, content, and expression methods, aiming to attract customers, increase purchase intentions, and promote sales.
(2) Core Elements and Theoretical Model: Based on grounded theory, the core elements of livestream marketing discourse are summarized, and a theoretical logical model is constructed to clarify the relationships among these elements. Livestream marketing discourse includes four key elements: product image portrayal, emphasis on service guarantees, customer interaction, and operational management during the livestream. Among these, product image portrayal is the core of marketing discourse; emphasizing service guarantees and customer interaction are essential support measures; and operational management during the livestream permeates the other three elements.

### 6.3. Contributions

*Theoretical Framework Development:* This study contributes to the understanding of live commerce marketing discourse by developing a comprehensive theoretical framework. By identifying and analyzing the core elements and their relationships, it provides a structured approach to examining the dynamics of live commerce interactions.

*Practical Insights for Anchors:* It offers practical guidance for anchors on how to effectively use marketing discourse to enhance product presentation, customer engagement, and operational efficiency. This can help anchors improve their strategies to attract and retain customers, ultimately leading to increased sales.

### 6.4. Recommendations for the development of livestream marketing discourse

This paper offers the following development recommendations for livestream marketing discourse:

(1) Highlight Practical and Hedonic Value: Anchors should innovate their marketing methods and focus on shaping their discourse identity, allowing consumers to perceive value while also experiencing emotional satisfaction, thereby enhancing their willingness to purchase
(2) Enhance Online Experience and Social Presence: anchors can construct experiential interactions from four dimensions: sensory, emotional, entertainment, and behavioral, which can lead to stronger evaluative concerns and higher information trust among consumers.
(3) Focus on Language Skills and Appropriateness: On the one hand, anchors should utilize effective language skills such as politeness, personalization, and rhetoric. On the other hand, anchors must adhere to ethical and moral standards.
(4) Multifaceted Regulation and Management: Governments should implement strict online governance regulations, encouraging livestream platforms to self-monitor and manage rigorously. Livestream platforms must assume corporate social responsibility, promoting mainstream values and positive energy. Anchors should combine personal interests with social responsibility, ensuring their discourse is meaningful, informative, and positive [65].

### 6.5. Research limitations and prospects

This study also has several limitations. First, the study employed a qualitative research method to identify the key elements and theoretical logic of live commerce marketing discourse. Although we strictly adhered to the operational requirements of grounded theory, there is inevitably some subjectivity involved. In the future quantitative research can test the generalizability and applicability of the findings. Second, the study only analyzed the composition elements and logical system of live commerce marketing discourse from the perspective of content elements, without considering non-verbal cues such as actions, expressions, and tone. Third, according to the current development of live commerce, the samples were mainly collected from China. Although the study covered a variety of products and considered differences in anchors identity and gender, this inevitably affects the applicability of the results. As live commerce rapidly develops globally, research considering different cultural and economic backgrounds can be explored in the future.

## References

[1] Shankar V, Grewal D, Sunder S, Fossen B, Peters K, Agarwal A. Digital marketing communication in global marketplaces: A review of extant research, future directions, and potential approaches. International Journal of Research in Marketing. 2022;39(2):541–65. doi: 10.1016/j.ijresmar.2021.09.005

[2] Sun Y, Shao X, Li X, Guo Y, Nie K. How live streaming influences purchase intentions in social commerce: An IT affordance perspective. Electronic Commerce Research and Applications. 2019;37:100886. doi: 10.1016/j.elerap.2019.100886

[3] Research team of the Institute of Finance and Trade Economics of the Chinese Academy of Social Sciences, the China Market Society. 2024 Live E-commerce Industry Development Trend Observation. In: [Internet]. XINHUA FINANCE. 27Dec. 2024 [cited 1 Jan 2025]. Available: https://www.cnfin.com/hg-lb/detail/20241227/4165363_1.html

[4] Sharma R. Live Commerce Platform Market. In: [Internet].DATA INTELO. 21Feb.2024 [cited 1 Jan 2025]. Available: https://dataintelo.com/report/live-commerce-platform-market

[5] Ho C-I, Liu Y, Chen M-C. Factors Influencing Watching and Purchase Intentions on Live Streaming Platforms: From a 7Ps Marketing Mix Perspective. Information. 2022;13(5):239. doi: 10.3390/info13050239

[6] Han T, Han J, Liu J, Li W. Effect of emotional factors on purchase intention in live streaming marketing of agricultural products: A moderated mediation model. PLoS One. 2024;19(4):e0298388. doi: 10.1371/journal.pone.0298388 38558061 PMC10984517

[7] Ailawadi KL, Beauchamp JP, Donthu N, Gauri DK, Shankar V. Communication and Promotion Decisions in Retailing: A Review and Directions for Future Research. Journal of Retailing. 2009;85(1):42–55. doi: 10.1016/j.jretai.2008.11.002

[8] Xu XY, Wu JH, Li Q. What drives consumer shopping behavior in live streaming commerce? Journal of electronic commerce research. 2020;21(3):144–67. http://ojs.jecr.org/jecr/sites/default/files/2020vol21no3_Paper1.pdf

[9] Zhang Y. Taobao Anchors Strategically Promote Impulsive Buying of Consumer. In: Proceedings of 2021 3rd International Conference on Economic Management and Cultural Industry; Guangzhou, China. 2021 Oct 22-24. p. 3027−3030. 10.2991/assehr.k.211209.494

[10] Liu Z, Yap TT, Zhang X, Sinayah M. What Do We Know about E-commerce Live Streaming Discourse from Five Years of Research? A Systematic Review (2019–2023). Forum Linguist Stud. 2024;6(6):767–84. doi: 10.30564/fls.v6i6.7520

[11] Huang HY, Blommaert J, Van Praet E. “OHMY GOD! BUY IT!” A Multimodal Discourse Anal-ysis of the Discursive Strategies Used by Chinese Ecommerce Live-Streamer Austin Li. In: Proceedings of the HCI Inter- national 2020–Late Breaking Papers: Interaction, Knowledge and Social Media: 22nd HCI Internation-al Conference; Copenhagen, Denmark; 2020 Jul 19-24. p. 305−27. doi: 10.1007/978-3-030-60152-2_24

[12] Song Z, Liu C, Shi R. How Do Fresh Live Broadcast Impact Consumers’ Purchase Intention? Based on the SOR Theory. Sustainability. 2022;14(21):14382. doi: 10.3390/su142114382

[13] Lakhan GR, Ullah M, Channa A, Abbas M, Khan MA. Factors effecting consumer purchase intention: live streaming commerce. Psychology and Education. 2021;58(5):601–11. doi: 10.1007/978-3-030-60152-2_24

[14] Cui F. Analysis and evaluation of relevant influencing factors based on the big data of Douyin live broadcast sales. J Phys Conf Ser. 2021;1955(1):012034. doi: 10.1088/1742-6596/1955/1/012034

[15] Gao YB, Liu SB. “Just Buy It!” The Discipling Force of Online Marketing:Discourse Analysis on Online Living Broadcasting. Journal of Central University of Finance & Economics. 2022;(12):134–47. doi: 10.19681/j.cnki.jcufe.2022.12.011

[16] Huang JH, Yang L. “True Feelings” or “False Intentions”: A Study on the Construction of Parasocial Relationships by Elderly Online Hosts. New Media Research. 2023;(01):73–7. doi: 10.16604/j.cnki.issn2096-0360.2023.01.025

[17] Chen Y. Culture Sells: Analysing New Ori-ental’s Live-streaming Communication Strategy from A Framing Perspective. In: Proceedings of the International Conference on Media Science and Digital Communication; Sri Lanka. 2022 Dec 3-4. p.18–28. doi: 10.17501/29506530.2023.2102

[18] Yang Y. Research on the Impact of Live Video Streaming on Customers’ Consumption Behavior and Intention. In: Proceedings of the 6th International Conference on Economics, Management, Law and Education (EMLE 2020); Krasnodar, Russia. 2020 Oct 29-30. p. 301–5. doi: 10.2991/aebmr.k.210210.048

[19] Wu KJ, Xu IL, Yan AH. Conversations in Live Streaming Selling: A Case Study in China. International Journal of Linguistics, Literature and Translation. 2022;5(10):11–7. doi: 10.32996/ijllt.2022.5.10.2

[20] Wongsunopparat S, Deng B. Factors Influencing Purchase Decision of Chinese Consumer under Live Streaming E-Commerce Model. Journal of Small Business and Entrepreneurship. 2021;9(2):1–15. doi: 10.15640/jsbed.v9n2a1

[21] Fu YY. Live Streaming Commerce: A Review and Prospects. In: Proceedings of the 2021 3rd International Conference on Economic Management and Cultural Industry (ICEMCI 2021); Guangzhou, China. 2021 Oct 22-24. p. 2546–52. doi: 10.2991/assehr.k.211209.414

[22] Lu B, Chen Z. Live streaming commerce and consumers’ purchase intention: An uncertainty reduction perspective. Information & Management. 2021;58(7):103509. doi: 10.1016/j.im.2021.103509

[23] Liu X, Zhang L, Chen Q. The effects of tourism e-commerce live streaming features on consumer purchase intention: The mediating roles of flow experience and trust. Front Psychol. 2022;13:995129. doi: 10.3389/fpsyg.2022.995129 36092030 PMC9462463

[24] Cai J, Wohn DY, Mittal A, Sureshbabu D. Utilitarian and Hedonic Motivations for Live Streaming Shopping. Proceedings of the 2018 ACM International Conference on Interactive Experiences for TV and Online Video. 2018. 81–8. doi: 10.1145/3210825.3210837

[25] Lu CY, Marek MW, Chen BT, Pai IC. An exploratory study on consumer purchase behavior from live webcasting e-commerce: A means-end chain analysis using Facebook live webcasting. International Journal of Online Marketing. 2020;10(3):1–20. doi: 10.4018/ijom.2020070101

[26] Qiu Q, Zuo Y, Zhang M. Can Live Streaming Save the Tourism Industry from a Pandemic? A Study of Social Media. IJGI. 2021;10(9):595. doi: 10.3390/ijgi10090595

[27] Huang HY, Blommaert J, Van Praet E. “OH MY GOD! BUY IT!” a multimodal discourse analysis of the discursive strategies used by Chinese ecommerce live-streamer Austin Li. In: Proceedings of the HCI International 2020–Late Breaking Papers: Interaction, Knowledge and Social Media: 22nd HCI International Conference; Copenhagen, Denmark; 2020 Jul 19-24. p. 305–27. doi: 10.1007/978-3-030-60152-2_24

[28] Song Y, Mo J. Discourse analysis of knowledge-based live streaming: a case study of East Buy streamer Dong Yuhui. Language and Semiotic Studies. 2024;10(2):222–44. doi: 10.1515/lass-2024-0017

[29] Gao X, Xu X-Y, Tayyab SMU, Li Q. How the live streaming commerce viewers process the persuasive message: An ELM perspective and the moderating effect of mindfulness. Electronic Commerce Research and Applications. 2021;49:101087. doi: 10.1016/j.elerap.2021.101087

[30] Research on the Influence Mechanism of Taobao Live Streaming Marketing Discourse on Consumer Behavior Tendency—Based on Demonstration of the ABC Attitude Model. MOS. 2023;12(05):4631–41. doi: 10.12677/mos.2023.125422

[31] Chen J, Zhou T. Antecedents of Consumers’ Impulsive Buying Intention in Live Streaming Commerce——Perspective of Live Streamer’s Persuasive Ability. In: Proceedings of the WHICEB 2022; Wuhan, China. 2022 May 27-29; https://aisel.aisnet.org/whiceb2022/26/

[32] Iqbal H, Asghar Z, Khan NW. Linguistic Politeness in the Marketing Discourse of Pushtoon Service Providers. Sjesr.2020; 3(3):309–17. doi: 10.36902/sjesr-vol3-iss3-2020(309-317)

[33] Barmina EA, Mestanko NA, Skidan OG. Creolized texts used as the instrument of digital marketing. In: Proceedings of the 1st International Scientific Conference“ Modern Management Trends and the Digital Economy: from Regional Development to Global Economic Growth”(MTDE 2019). Yekaterinburg, Russia. 2029 Apr 14-15. p. 525-528. doi: 10.2991/mtde-19.2019.105

[34] Murashova EP. The role of the cognitive metaphor in the hybridisation of marketing and political discourses: An analysis of English-language political advertising. TLC. 2021;5(2):22–36. doi: 10.22363/2521-442x-2021-5-2-22-36

[35] Yang N, Wang ZH. Addressing as a Gen-der-Preferential Way for Suggestive Selling in Chinese E-Commerce Live Streaming Discourse: A Corpus-Based Approach. Journal of Pragmatics. 2022;197:43−54. doi: 10.1016/j.pragma.2022.05.014

[36] Zhang G, Hjorth L. Live-streaming, games and politics of gender performance: The case of Nüzhubo in China. Convergence: The International Journal of Research into New Media Technologies. 2017;25(5–6):807–25. doi: 10.1177/1354856517738160

[37] Wang YC, Zeng Y. Research on Discourse Applicability in Network Broadcast Marketing: Based on Grounded Theory Research Method. Journal of Xiangtan University (Philosophy and Social Sciences). 2022;46(02):188–93. doi: 10.13715/j.cnki.jxupss.2022.02.030

[38] Aziz SAH, Othman SK. Speech acts uses in persuasion and deception in marketing discourse. Journal of University of Babylon for Humanities. 2020;28(6):73–62. https://www.iraqoaj.net/iasj/download/bb4b633a9391e1ab

[39] Abdumutaljonovna PS. Main Characteristics of Advertising Discourse in Modern Linguistics. Texas Journal of Multidisciplinary Studies. 2022;9:173–76. https://zienjournals.com/index.php/tjm/article/view/2033

[40] Fitchett J, Caruana R. Exploring the role of discourse in marketing and consumer research. J of Consumer Behaviour. 2014;14(1):1–12. doi: 10.1002/cb.1497

[41] Corbin JM, Strauss A. Grounded theory research: Procedures, canons, and evaluative criteria. Qual Sociol. 1990;13(1):3–21. doi: 10.1007/bf00988593

[42] Siggelkow N. Persuasion With Case Studies. Academy of Management Journal. 2007;50(1):20–4. doi: 10.5465/amj.2007.24160882

[43] Huang TY, Nie R, Zhao Y. Archival knowledge in the field of personal archiving: an exploratory study based on grounded theory. Journal of Documentation. 2020;77(1):19–40. doi: 10.1108/JD-04-2020-0071

[44] Glaser BG. Doing Grounded Theory: Issues and Discussions. Mill Valley, CA: Sociology Press; 1998.

[45] She SX, X HR, X DY. Psychological contract between consumers and anchors in live marketing: scale development and its dynamic evolution. Journal of Finance and Economics. 2022;(06):93–102. doi: 10.13762/j.cnki.cjlc.2022.06.003

[46] Liu JY, Sun HY, Li JF, Chen R, Zhang Y, Dong QX. Research on factors affecting consumer purchase intention in e-commerce live broadcast - based on the dual path model perspective. Journal of Chongqing University of Arts and Sciences (Social Sciences Edition). 2024;(01):49–60. doi: 10.19493/j.cnki.issn1673-8004.2024.01.006

[47] Chen YT, Zhao JW, Yuan SJ. The evolution mechanism from psychological contract to consumption intention in e-commerce live broadcast - the moderating role of involvement. China Circulation Economy. 2021;(11):44–55. doi: 10.14089/j.cnki.cn11-3664/f.2021.11.005

[48] Xiong X, Zhu CX, Zhu HB. The formation mechanism of consumer trust in agricultural products e-commerce live broadcast: from the perspective of intermediary capacity. Journal of Nanjing Agricultural University (Social Sciences Edition). 2021;(04):142–54.42. doi: 10.19714/j.cnki.1671-7465.2021.0064

[49] Ye C, Zheng R, Li L. The effect of visual and interactive features of tourism live streaming on tourism consumers’ willingness to participate. Asia Pacific Journal of Tourism Research. 2022;27(5):506–25. doi: 10.1080/10941665.2022.2091940

[50] Shang Q, Ma H, Wang C, Gao L. Effects of Background Fitting of e-Commerce Live Streaming on Consumers’ Purchase Intentions: A Cognitive-Affective Perspective. Psychol Res Behav Manag. 2023;16:149–68. doi: 10.2147/PRBM.S393492 36699986 PMC9869996

[51] Horton D, Richard Wohl R. Mass communication and para-social interaction: Observations on intimacy at a distance. Psychiatry. 1956;19(3):215–29. doi: 10.1007/978-3-658-09923-7_7 13359569

[52] Xie Y, Li C, Gao P, Liu Y. The effect and mechanism of social presence in live marketing on online herd consumption from behavioral and neurophysiological perspectives. Adv Psychol Sci. 2019;27(6):990–1004. doi: 10.3724/sp.j.1042.2019.00990

[53] Yuan DH, Gao LD. Para-social interaction in social media and its marketing effectiveness. Foreign Economics & Management. 2020;42(7):21–35. doi: 10.16538/j.cnki.fem.20200407.301

[54] Peng YH, Han H, Hao LG, Huo JL, Wang YD. Research on the influence of relationship ties and customer commitment on consumers’ online purchase intention in live streaming marketing. Chinese Journal of Management. 2021;(11):1686–94. doi: 10.3969/j.issn.1672-884x.2021.11.012

[55] Goffman E. The Interaction Order: American Sociological Association, 1982 Presidential Address. American Sociological Review. 1983;48(1):1. doi: 10.2307/2095141

[56] Chen Y, Lu F, Zheng S. A Study on the Influence of E-Commerce Live Streaming on Consumer Repurchase Intentions. International Journal of Marketing Studies. 2020;12(4):48. doi: 10.5539/ijms.v12n4p48

[57] Pozharliev R, Verbeke WJMI, Van Strien JW, Bagozzi RP. Merely Being with you Increases My Attention to Luxury Products: Using EEG to Understand Consumers’ Emotional Experience with Luxury Branded Products. Journal of Marketing Research. 2015;52(4):546–58. doi: 10.1509/jmr.13.0560

[58] Han YT, Zhou Jl, Ren F. A Dynamic Perspective on the Impact of Live Comments Content on Product Sales of Live Streaming Commerce. Journal of Management Science. 2022;(01):17–28. doi: 10.3969/j.issn.1672-0334.2022.01.002

[59] Guo H, Sun X, Pan C, Xu S, Yan N. The Sustainability of Fresh Agricultural Produce Live Broadcast Development: Influence on Consumer Purchase Intentions Based on Live Broadcast Characteristics. Sustainability. 2022;14(12):7159. doi: 10.3390/su14127159

[60] Hansen T, Møller Jensen J, Stubbe Solgaard H. Predicting online grocery buying intention: a comparison of the theory of reasoned action and the theory of planned behavior. International Journal of Information Management. 2004;24(6):539–50. doi: 10.1016/j.ijinfomgt.2004.08.004

[61] Foucault M. panopticism“ from” discipline & punish: The birth of the prison. Race/Ethnicity: multidisciplinary global contexts. 2008;2(1):1–12. http://www.jstor.com/stable/25594995

[62] Dong JJ, Xu ZL, Fang Q, Zhang AR. Model construction of the impact of online experiential interaction between consumers and merchants on their purchase intention. Chinese Journal of Management. 2018;(11):1722–30. doi: 10.3969/j.issn.1672-884x.2018.11.017

[63] Atasoy O, Morewedge CK. Digital Goods Are Valued Less Than Physical Goods. Journal of Consumer Research. 2017;44(6):1343–57. doi: 10.1093/jcr/ucx102

[64] Shi X, Dou H. How broadcasters enhance rapport with viewers in live streaming commerce. Pragmatics. 2022;33(4):592–617. doi: 10.1075/prag.22009.shi

[65] Yang L, Liu LL. Two-sided matching game between enterprises and anchors in the enterprise self-broadcasting model. Journal of Shandong University of Science and Technology (Social Sciences). 2024;26(04):99–112. doi: 10.16452/j.cnki.sdkjsk.2024.04.012

